# iAPF: an improved artificial potential field framework for asymmetric dual-arm manipulation with real-time inter-arm collision avoidance

**DOI:** 10.3389/frobt.2025.1604506

**Published:** 2025-10-28

**Authors:** S. K. Surya Prakash, Darshankumar Prajapati, Bhuvan Narula, Amit Shukla

**Affiliations:** 1 School of Mechanical and Materials Engineering, Indian Institute of Technology Mandi, Mandi, India; 2 Centre for Artificial Intelligence and Robotics, Indian Institute of Technology Mandi, Mandi, India; 3 School of Computing and Electrical Engineering, Indian Institute of Technology Mandi, Mandi, India

**Keywords:** collision avoidance, motion planning, improved artificial potential field, dual-arm manipulators, industrial automation

## Abstract

This paper presents a robust vision-based motion planning framework for dual-arm manipulators that introduces a novel three-way force equilibrium with velocity-dependent stabilization. The framework combines an improved Artificial Potential Field (iAPF) for linear velocity control with a Proportional-Derivative (PD) controller for angular velocity, creating a hybrid twist command for precise manipulation. A priority-based state machine enables human-like asymmetric dual-arm manipulation. Lyapunov stability analysis proves the asymptotic convergence to desired configurations. The method introduces a computationally efficient continuous distance calculation between links based on line segment configurations, enabling real-time collision monitoring. Experimental validation integrates a real-time vision system using YOLOv8 OBB that achieves 20 frames per second with 0.99/0.97 detection accuracy for bolts/nuts. Comparative tests against traditional APF methods demonstrate that the proposed approach provides stabilized motion planning with smoother trajectories and optimized spatial separation, effectively preventing inter-arm collisions during industrial component sorting.

## 1 Introduction

The increasing demand for industrial automation is driven by the need to enhance production speed, accuracy, and efficiency. Robots are increasingly replacing human workers to address challenges like fatigue and maintain consistent performance over extended periods (Liu et al., 2022). Despite challenges with human workers, bi-manual operations offer significant value through component recognition and manipulation. Humans instinctively understand unfamiliar objects by material, shape, size, and color, enabling effective planning for separation or assembly tasks. Integrating these capabilities into robotics is crucial for upgrading industrial automation through: i) HRC - Human robot collaboration where humans and robots collaboratively enhance process efficiency ii) robots with capabilities that mimic human intelligence and dexterity. A dual-arm system with efficient inter-arm collision avoidance and vision perception provides a robust solution matching human-like manipulations, essential for industrial component handling in warehouse management, assembly lines, and manufacturing.

In general, dual-arm manipulators offer improved dexterity and faster task completion compared to single arm manipulators. However, these advantages require careful consideration of movements to avoid inter-arm collisions while maintaining efficiency (Smith et al., 2012). Categories of dual-arm manipulators include the following: i) non-coordinated manipulations, where manipulators move independently executing different tasks, and ii) coordinated manipulations, involving combined movements with spatial and temporal synchronization in shared workspace. Coordinated manipulations further divide into symmetric (mirror movements with equal force distribution) and asymmetric (complementary movements with different force distributions) (Makris, 2021). Although more challenging, asymmetric manipulations enable a wider range of complex tasks that require coordinated actions. Integration with real-time vision and inter-arm collision awareness enhances robustness for industrial manufacturing assembly and warehouse management applications.

The planning of dual-arm movements addresses coordination through two primary approaches: path planning and motion planning. Path planning generates geometric trajectories avoiding obstacles and self-collisions, prioritizing spatial relationships without timing information. Motion planning extends this by incorporating velocity profiles, acceleration constraints, and dynamic considerations, creating time-parametrized trajectories (Abbas et al., 2023). The selection between these approaches depends on application requirements, with industrial manipulation tasks often benefiting from motion planning’s ability to manage the temporal aspects of coordination between manipulators. In addition Tang et al. (2012) provides a comprehensive review of robot motion planning approaches, categorizing and analyzing the research evolution from 1980 to 2010. They systematically compared conventional approaches like Bug Algorithms Lumelsky and Stepanov (1987), Roadmap Asano et al. (1985); Aurenhammer (1991); Canny (1989), Cell Decomposition, Potential Fields Khatib, (1986), and Mathematical Programming with heuristic methods (Neural Networks, Genetic Algorithms, Fuzzy Logic, etc.), demonstrating the field’s evolution from conventional methods in the 1980s to predominantly heuristic approaches by the 2000s. Their analysis includes quantitative comparisons of implementation frequency for each approach and identifies future research directions for integrating perception, sensing, and motion planning system provides useful insights for research directions.

## 2 Related work

Numerous studies enhanced dual-arm motion planning for industrial manipulation. Robust inter-arm collision avoidance improved collaborative motion by enabling safe execution of coordinated tasks. This capability required continuous awareness of relative link configurations, maintaining safe distances while maximizing shared workspace utilization. Real-time collision monitoring provided the foundation for effective dual-arm coordination in dynamic environments.

This literature review categorizes dual-arm manipulation research into three key areas: (i) collision detection methodologies (both geometric and alternative approaches), (ii) artificial potential field methods (ranging from classical formulations to specialized variants), and (iii) temporal coordination strategies. This organization traces the field’s evolution while revealing challenges in real-time adaptation and asymmetric manipulation challenges that suggest the potential value of extending APF approaches with improved force equilibrium mechanisms.

Effective dual-arm manipulation requires robust collision detection to prevent inter-arm collisions while maintaining operational efficiency. Researchers have developed various approaches, from geometric primitives to advanced mathematical frameworks, to address this fundamental challenge. Lumelsky (1985) introduced a parameter-based algorithm for minimum distance calculation between line segments in n-dimensional space. Using parameters t and u (range [0,1] for points inside segments), they established a framework handling all geometric configurations. The method first calculates minimum distance between infinite lines by minimizing a quadratic function, then verifies if these points lie within actual segments. By using parameter values to identify relevant endpoints, computational complexity reduces to 5n+12 multiplications and 8n+1 additions. However, it provides only discrete collision detection without continuous distance metrics, limiting its application in modern control approaches requiring gradient information for smooth motion planning. Furthermore, Chang et al. (1990) proposed collision detection approach uses minimum distance functions defined by Euclidean norms to determine collision risk between robot arms. Their method modeled robot links as cylinders with hemispheres and end-effector as spheres, then calculates distances between these primitives in three scenarios: line segment to line segment, line segment to point, and point to point. Lee and Moradi (2001) proposed a hierarchical geometric collision detection algorithm for dual-arms using: bounding boxes, joint-joint distance thresholds, perpendicular distance calculations, and cross-product methods. Despite a 30 % computational improvement, it used simplified primitives, lacked mathematical formalism for complex configurations, and provided only discrete binary detection without continuous metrics for smooth planning. The approach showed reduced accuracy with near-parallel links and increased complexity in 3-D without addressing degenerate cases or providing control gradient information. Ketchel and Larochelle (2008) presented a dual-stage collision detection algorithm for spatial closed chains first uses dual vector algebra and Plücker coordinates (Miller, 2005) to quickly check for possible collisions between infinite cylinders. If potential collisions are detected, a second stage performs precise testing between finite cylinders using detailed geometric calculations. While mathematically elegant, this approach may not provide the continuous distance metrics needed by modern control systems for obstacle avoidance. The method formulates collision detection as a constrained nonlinear minimization problem, which introduces complexity for real-time applications, requiring conjugate gradient methods with barrier functions to solve. Larsen et al. (2000) presented an RSS-based proximity algorithm that combines rectangles with uniform sphere expansion to create efficient bounding volumes. It builds hierarchical trees using covariance analysis and geometric “slab” properties for fast distance calculations. Performance improvements come from priority-directed search and triangle caching that leverages frame-to-frame coherence. While outperforming sphere-based hierarchies, the approach struggles with non-convex geometry (requiring decomposition into multiple parts) and can experience numerical instabilities in near-intersection cases, sometimes producing overly conservative distance estimates that reduce query efficiency. Rescsanski et al. (2025) provide insights into collaborative robotics for industrial additive manufacturing, highlighting effective collision detection methods using geometric approaches and real-time mechanisms for multi-arm systems. It analyzes motion planning strategies for cooperative robotic systems where coordination between arms is crucial for manufacturing quality and efficiency. The research presents a framework for cooperative robotic arm additive manufacturing (C-RAAM) configurations based on build volume overlap, enabling applications from large-scale homogeneous printing to specialized heterogeneous tooling. It demonstrates how inter-arm coordination and collision avoidance can be achieved through either preventative scheduling or real-time sensing and feedback.

Apart from the geometric approaches, researchers explored alternative methods for collision detection for dual-arm collaboration. Feng et al. (2020) introduced a graph-based network approach for multi-robot collision detection where robots are represented as vertices with edges connecting interacting robots. It focuses on maintaining network connectivity through local and global edge management methods, using algebraic connectivity measures and market-based algorithms. Limitations include computational demands from continuous eigenvalue calculation, potential delays in link decisions, and the focus on network connectivity potentially overlooking actual collisions, which may compromise safety. Jamisola and Roberts (2015); Jamisola et al. (2015) presented a transformation-based approach to multi-manipulator kinematics that focuses on relative motion between end-effectors while providing a mathematical framework for coordinate transformations relevant to absolute motion control. They express individual manipulator Jacobians with rotation and wrench transformation matrices to ensure coordinate consistency across the system. Their analysis shows that positional errors increase significantly at higher angular velocities when transformation matrices are incomplete, emphasizing the need for rigorous mathematical formulations when multiple manipulators share a workspace. This approach complements geometric collision detection by providing insight into how manipulator kinematics must be properly transformed between reference frames for accurate motion planning. Fei et al. (2004) presented a dual-arm collision avoidance approach using configuration space (C-Space) representation. The methodology identifies collision regions through reachable manifold and contact manifold concepts, then applies the A* algorithm on discretized grids to find optimal paths. Path optimization is incorporated through scan rules. While innovative for its time, the approach has limitations: it relies on static geometric planning rather than continuous adaptation, uses discretized boundaries that may limit motion smoothness, and lacks mechanisms for maintaining optimal arm configurations during complex movements. Schmidt and Wang (2014) implemented collision avoidance using Kinect depth sensors that process images by removing backgrounds and converting obstacles to 3-D point clouds with efficient bounding box representation. Their system calculates minimum distances between robot and obstacles, enabling three strategies: warnings, stops, or path modification through vector decomposition. This methodology provided valuable insights for our vision perception conceptualization, particularly in how sensor data can be transformed into actionable spatial information for real-time goal attraction with collision avoidance in robot-robot collaborative environments.

Physics-inspired potential field approaches offer an intuitive framework for robot motion planning by simulating attractive and repulsive forces. Beginning with Khatib’s foundational work Khatib (1986), these methods have evolved to address limitations like local minima while accommodating increasingly complex manipulation tasks. Khatib (1986) pioneered the Artificial Potential Field (APF) method, transforming discrete path planning into a continuous optimization problem. The robot is treated as a particle moving in a force field where an attractive force proportional to goal distance pulls the robot toward the target, while repulsive forces decaying quadratically with proximity push it away from obstacles. The robot follows the negative gradient of this composite potential function, implementing steepest descent optimization in configuration space. A key limitation is that APF can suffer from local minima in cluttered environments due to the superposition of attractive and repulsive forces. Subsequent research by Volpe and Khosla (1987), Volpe and Khosla (1990) refined the original APF concept, by introducing n-ellipse representation that varies with distance. The n-elliptical iso-potential contours address the fundamental limitations in obstacle avoidance. Their innovative approach seamlessly transitioned from rectangular contours near obstacles (n→∞) to circular fields at greater distances (n→1) , effectively eliminating local minima through geometric adaptation. While this method significantly improved single-arm manipulation, its computational complexity in calculating elliptical potentials limited real-time applications. Furthermore, Chuang et al. (2006) proposed a dual-arm coordination strategy employing APF was proposed. This approach uses an alternating master-slave paradigm. Initially, one arm (the master) plans its trajectory while treating the other arm as a static obstacle. Subsequently, the roles are reversed, with the previously slave arm now planning its trajectory while the other arm remains static. This alternating priority scheme continues until all objects reach their respective goals. The workspace is modeled using charged surfaces that generate repulsive forces to ensure avoidance of collisions between robots and obstacles. Byrne et al. (2015) and Byrne et al. (2013) enhanced APF by integrating configuration sampling and sub-goal selection within a master-slave architecture and cooperative goal sampling framework. This approach achieved a 95.2% success rate, demonstrating its effectiveness in handling more complex coordinated tasks. However, it comes at the cost of increased computational overhead and slower convergence rates (9.14s vs. 4.65s) compared to the original APF (Khatib, 1986). Additionally, its application is limited to planar 2D manipulation scenarios.

Furthermore, enhanced APF with specialized variants, such as D-APF(Dynamic Artificial Potential Field) Jayaweera and Hanoun (2020) for UAV path planning using exponential attractive and repulsive potential fields for moving target tracking and obstacle avoidance. It prioritizes vertical over horizontal motion, favoring altitude changes for obstacle avoidance. Combined forces generate smooth trajectories converted to velocity waypoints for PX4 autopilot, with separate PD-based orientation control. The force separation into vertical/horizontal components adds complexity and may cause oscillations during rapid directional changes, and the approach struggles with complex or closely spaced obstacles where vertical avoidance is insufficient. D-APF demonstrates potential field method evolution for aerial navigation, though dual-arm manipulation presents unique challenges requiring careful force field design. Sun et al. (2022) used APF in a hybrid approach with their mobile dual-arm system combined with RRT (Rapidly Exploring Random Tree)-based motion planning for base navigation along with integrated vision and impedance control. Wang et al. (2018) developed an optimized APF with velocity feedforward and simplified collision detection for redundant manipulators. Li et al. (2024a) and Li et al. (2024b) introduced an attractive potential field rotation method for local minima avoidance and develop a smooth attractive function σ(d) to address Goals Non-Reachable with Obstacles Nearby (GNRON) issues. While effective for single-robot navigation, the approach is limited to navigation rather than manipulation scenarios. Despite mathematical elegance, it faces constraints when extended to multi-robot coordination and precision control in healthcare environments. Their rotation-based solution introduces computational considerations that must be balanced against real-time performance requirements in practical applications. Lin et al. (2025) proposed a 3-D APF approach for robotic arms that integrates force sensing with rotating repulsive fields to navigate narrow spaces. Their method treats the tool as a vector rather than a point and modifies repulsive force direction to prevent local minima. While their approach addressed single-arm collision avoidance, our method extends APF to dual-arm coordination through a novel three-way force equilibrium with exponential home-seeking forces and priority-based state transitions that enable asymmetric dual-arm manipulation with proven stability.

Beyond spatial collision avoidance, successful dual-arm manipulation demands sophisticated temporal coordination to synchronize movements and optimize task execution. Researchers have developed various frameworks to address this critical dimension of collaborative robotic systems. Montaño and Suárez (2016) proposed a temporal coordination approach for multiple robots using Discretized Coordination Space (DCS) where robots adjust timing along fixed paths. It builds a Collision-free Coordination Curve using state diagrams with wall-follower or impact heuristics to select motion directions. The computational complexity increases exponentially with robot numbers ( $3^{n}-1$ motion directions), lack of flexibility for dynamic environments since paths cannot be modified, and frequent robot idle periods when coordination points cannot be generated quickly enough, reducing system efficiency compared to reactive approaches like iAPF. Malvido Fresnillo et al. (2023) developed an automatic tool change system integrated with MoveIt’s scene management, allowing robots to exchange end effectors by manipulating collision objects and communicating with hardware interfaces. For dual-arm coordination, they created synchronization methods that merge individual arm trajectories using various timing policies, enabling coordinated manipulation through time-adjusted motion plans or master-slave relationships. However, this planning-based approach has inherent limitations, it requires complete environment knowledge beforehand, and it lacks the reactivity needed for dynamic obstacle avoidance or real-time adaptation to changing conditions. Shin and Bien (1989) presented a collision-free trajectory planning approach for two robotic arms sharing a workspace by transforming obstacles into a virtual coordination space (VCS). Their method allows links of one robot to grow while shrinking the other to a point, visualizing all collision-free coordinations as curves in the VCS. Although innovative for dual-arm coordination, their approach requires explicit modeling of virtual obstacles and lacks the dynamic adaptability of our three-way force equilibrium system that maintains optimal arm configurations throughout the workspace. Basile et al. (2012) proposed a task-oriented motion planning framework that enables programming at the workpiece level through a cooperative planner handling task-space trajectories and kinematic transformations, while separate arm planners compute inverse kinematics for each robot. Despite efficient coordination through their taxonomy-based approach (distinguishing positioners and workers) and extended programming language with high-level instructions, their method suffers from limited real-time adaptability, as it requires complete task specification beforehand and cannot dynamically respond to unexpected obstacles or environmental changes, unlike our iAPF method, which continuously recalculates forces to generate reactive behaviors that smoothly adapt to changing conditions without extensive reprogramming.

Furthermore, for effective object handling in real-time operation, precise detection of required components is an important aspect. Object detection has evolved from traditional computer vision techniques like SIFT, SURF, and BRIEF -which require manual feature extraction and parameter tuning O’Mahony et al. (2019) to more advanced CNN methods that automatically learn relevant features (Alzubaidi et al., 2021). For handling industrial components, an accurate pose estimation is crucial. K et al. (2024) presented a classical approach for bolt handling using k-mean clustering and chamfer matching, offering simple implementation without training data but limited by sensor quality and lighting conditions. S. K et al. (2024) employed DeeplabV3P with classical techniques for estimating and sorting bolt size. Instance segmentation provides both classification and precise localization with clean edge masks (Sharma et al., 2022). Mask R-CNN He et al. (2017), built on Faster R-CNN Ren (2015) which uses region proposal networks instead of selective search Girshick (2015); Girshick et al. (2014) adds a parallel branch for segmentation masks. Detectron2 Wu et al. (2019), successor to Detectron Girshick et al. (2018) and maskrcnn-benchmark Massa and Girshick (2018), incorporates Mask R-CNN and RetinaNet with focal loss. For real-time inference on CPU machines, Ultralytics’ YOLOv8 and YOLOv11 Jocher et al. (2023); Jocher and Qiu (2024) offer faster performance through single-pass architecture, lightweight backbones, anchor-free detection, and efficient feature pyramids. YOLOv8’s oriented bounding boxes capability avoids computationally expensive instance segmentation.

Our improved Artificial Potential Field (iAPF) approach addresses key limitations identified across existing methodologies as shown Table 1. While geometric approaches like Lumelsky (1985) and Chang et al. (1990) provide precise collision detection, they lack continuous distance metrics for smooth motion planning. Traditional APF methods by Khatib (1986) offer intuitive frameworks but suffer from local minima, particularly in cluttered environments. Prior coordination strategies like Montaño and Suárez (2016), discretized coordination space and Malvido Fresnillo et al. (2023), master-slave paradigm lack dynamic adaptability to changing environments. iAPF overcomes these limitations through a novel three-way force equilibrium that balances goal attraction, obstacle repulsion, and home-seeking behavior. Unlike Byrne et al. (2015) and Byrne et al. (2013) approach, which showed slower convergence despite higher success rates, our method achieves real-time performance without sacrificing stability. Where Li et al. (2024a) and Li et al. (2024b) rotating repulsive fields work well for single arms, our approach extends to dual-arm coordination with a priority-based state transition mechanism that enables truly asymmetric dual-arm manipulation without oscillations or deadlocks. By addressing the need for continuous adaptation rather than pre-planned trajectories, iAPF motion planning along with real-time 20 frames per second (FPS) inference provides a robust solution for collaborative robot tasks in dynamic industrial environments like manufacturing, warehouse management and assembly line fields. Our novel iAPF methodology addresses these limitations through the following key contributions.

1. Case-Specific Analytical Inter-Arm Distance - Algorithm employing closed-form vector solutions for parallel, intersecting, and skew link configurations with epsilon-based numerical stability handling.
2. Hybrid Linear-Angular Control Framework - iAPF-derived linear velocity control coupled with independent PD (Proportional Derivative)-regulated angular velocity, creating a dual-control system with proven Lyapunov stability for exponential $\left(O\left(e^{d}\right)\right)$ attractive and inverse power-law $\left(O\left(d^{-2.3}\right)\right)$ repulsive force integration.
3. 4-DoF Vision-Based Pose Estimation - Real-time component detection (20 FPS) with oriented bounding box analysis providing position and yaw for precise grasp planning.
4. State-Conditional Kinodynamic Control - Velocity damping coefficients and task-specific z-axis constraints adaptively modulated based on operational phase (targeting, gripping, transport).
5. Priority-Based Dual-Arm Task Allocation - Hierarchical state machine with comparative-distance-based resource locking for deadlock-free simultaneous manipulation in shared workspaces.

**TABLE 1:** Comparative analysis of the proposed iAPF with traditional and existing APF variants for industrial dual-arm manipulation.

Method	Force field components	Stability guarantee	Real-time performance	Industrial suitability	Key industrial limitations
Traditional APF (Khatib, 1986)	Attractive + Repulsive	None	Moderate	Low	Local minima in cluttered workspaces
Enhanced APF (Byrne et al., 2015)	Attractive + Repulsive + Sampling	Statistical (95.2%)	Slow (9.14s)	Moderate	High computational overhead
D-APF (Jayaweera and Hanoun, 2020)	Exponential + Vertical Priority	None	Moderate	Low	Limited to UAV applications
Rotating APF (Lin et al., 2025)	Attractive + Rotating Repulsive	Mathematical	Moderate	Low	Single arm only, no coordination
iAPF (Proposed)	Attractive + Repulsive + Home-seeking	Lyapunov Proven	Fast (10 Hz)	High	Dynamic obstacle adaptation

This paper is organized as follows: Section 1 introduces the motivation and significance of asymmetric bi-manual manipulations in industrial automation. Section 2 provides a systematic review of relevant literature, highlighting the advantages of our proposed method. Section 3 presents the proposed methodology for establishing inter-arm collision avoidance in dual-arm motion planning, incorporating real-time vision and providing theoretical proofs for the potential field formulations. Section 4 describes the experimental setup, including the hardware used and the required transformation of frames. Section 5 presents a comparative analysis between the proposed iAPF and traditional APF methods, highlighting the advantages of our approach, and showcases the deep learning model’s performance metrics. Finally, Section 6 summarizes the key findings, presents the conclusions, and discusses potential future research directions.

## 3 Methodology

**FIGURE 1:**
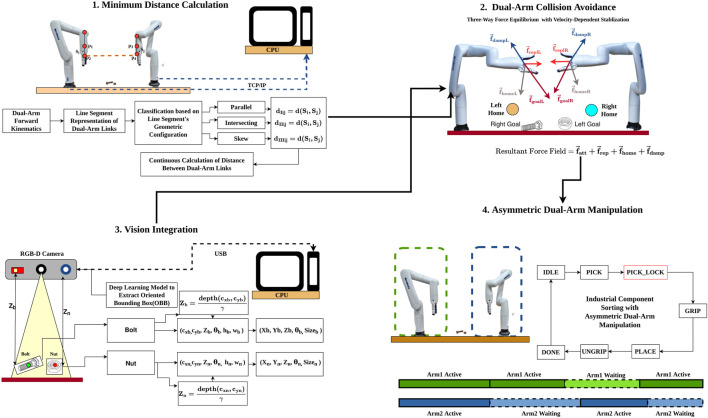
The Proposed Overall Methodology of iAPF framework for Asymmetric Dual-Arm Manipulation with Real-Time Inter-Arm Collision Avoidance.

The proposed overall methodology as shown in Figure 1 integrates four key components: (1) minimum distance calculation between inter-arm links using geometric classification, (2) vision-based pose estimation for industrial components, (3) three-way force equilibrium combining attractive, repulsive, and home-seeking forces with velocity-dependent stabilization and (4) state-based asymmetric coordination for dual-arm manipulation with priority locking. Together, these components enable collision-free, coordinated manipulation in shared workspaces.

### 3.1 Minimum distance calculation between inter-arm links using geometric classification

**FIGURE 2:**
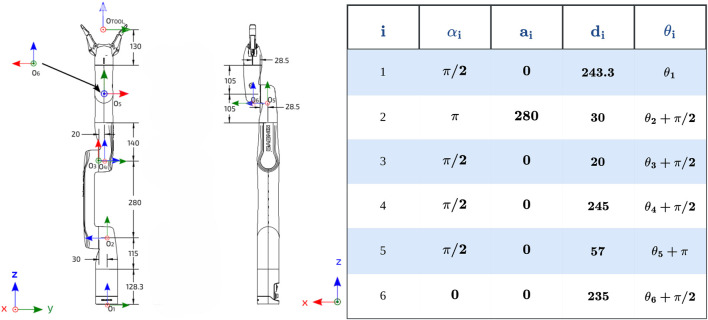
Kinova Gen3 lite with link frames with modified DH parameters for forward kinematics computation.

We modeled each robotic arm link as a line segment in 3-D space using Equation 1 and Equation 2 as given in Equation 3 using D-H parameters shown in Figure 2 enabling efficient minimum distance calculation between inter-arm links through geometric classification as parallel, intersecting, or skew configurations.

$$T_{i,k}^{i-1}=\left[\begin{array}{cccc} \cos\left(\theta_{i}\right) & -\cos\left(\alpha_{i}\right)\sin\left(\theta_{i}\right) & \sin\left(\alpha_{i}\right)\sin\left(\theta_{i}\right) & a_{i}\cos\left(\theta_{i}\right)\\ \sin\left(\theta_{i}\right) & \cos\left(\alpha_{i}\right)\cos\left(\theta_{i}\right) & -\sin\left(\alpha_{i}\right)\cos\left(\theta_{i}\right) & a_{i}\sin\left(\theta_{i}\right)\\ 0 & \sin\left(\alpha_{i}\right) & \cos\left(\alpha_{i}\right) & d_{i}\\ 0 & 0 & 0 & 1 \end{array}\right]$$ (1)

$$T_{i,k}^{i-1}=\left[\begin{array}{cc} R_{i,k}^{i-1} & p_{i,k}^{i-1}\\ 0 & 1 \end{array}\right]$$ (2)

$$S_{i,k}=p_{i,k}^{i-1}$$ (3)

$$T_{\mathrm{tool,k}}^{0}=T_{1,k}^{0}\cdot T_{2,k}^{1}\cdot T_{3,k}^{2}\cdot T_{4,k}^{3}\cdot T_{5,k}^{4}\cdot T_{6,k}^{5}\cdot T_{\mathrm{tool,k}}^{6}$$ (4)

Where i=1…6 represents the joint number, tool represents the end-effector frame, k denotes the arm identifier (right/left arm), $R_{i,k}^{i-1}\in R^{3\times 3}$ is the rotation matrix, and $p_{i,k}^{i-1}\in R^{3}$ is the position vector.

Theorem 1 (Segment Properties). *Every line segment* S *possesses, convexity:* ∀x,y∈S,λx+(1−λ)y∈S *for* λ∈[0,1] *, compactness of* S *is closed and bounded, connectedness of* S *is path-*connected (Munkres, 2000).

The segment between two bounding points $p_{1},p_{2}$ is $S\left[p_{1},p_{2}\right]$ , direction vector $\vec{d}=p_{2}-p_{1}$ , unit direction $\hat{d}=\frac{\vec{d}}{\left\|\vec{d}\right\|}$ , and normal space is $N=\left\{v\in R^{3}|v\cdot \hat{d}=0\right\}$ . The normal space N is a two-dimensional subspace of $R^{3}$ (O’neill, 2006).

For segments $S_{1},S_{2}$ with direction vectors $\vec{d_{1}},\vec{d_{2}}$ , then the angle between them is:

$$\theta =\arccos\left(\frac{\vec{d_{1}}\cdot \vec{d_{2}}}{\left\|\vec{d_{1}}\|\|\vec{d_{2}}\right\|}\right)$$

Theorem 2 (Angle Invariance). The angle θ between segments is invariant under rigid body transformations, such as translation and rotation (Gallier, 2011).

Theorem 3 (Configuration Completeness). *Every pair of line segments falls exactly into one of:*

1. *Intersecting*
2. *Parallel*
3. *Skew*

(Pottmann et al., 2001).

Theorem 4 (Metric Space Properties). *The function* d *satisfies, positive definiteness* d(x,y)≥0 *,* d(x,y)=0 *when* x=y *, symmetry* d(x,y)=d(y,x) *and triangle inequality* d(x,z)≤d(x,y)+d(y,z) *. Furthermore,* $\left(R^{3},d\right)$ *is a complete metric space.*

The distance function d is continuous in both arguments if and only if.

For all ϵ>0 , there exists a δ>0 such that if $\|x_{1}-x_{2}\|<\delta$ and $\|y_{1}-y_{2}\|<\delta$ , then $|d\left(x_{1},y_{1}\right)-d\left(x_{2},y_{2}\right)|<\epsilon$ .

The distance between a line segment on the left arm $S_{i\text{left}}$ and a line segment on the right arm $S_{i\text{right}}$ is defined as:

$$d\left(S_{i\text{left}},S_{i\text{right}}\right)=\min\|x-y\|$$ (5)

where, $x\in S_{i\text{left}},\;y\in S_{i\text{right}}\;\in R^{3}$ .

**FIGURE 3:**
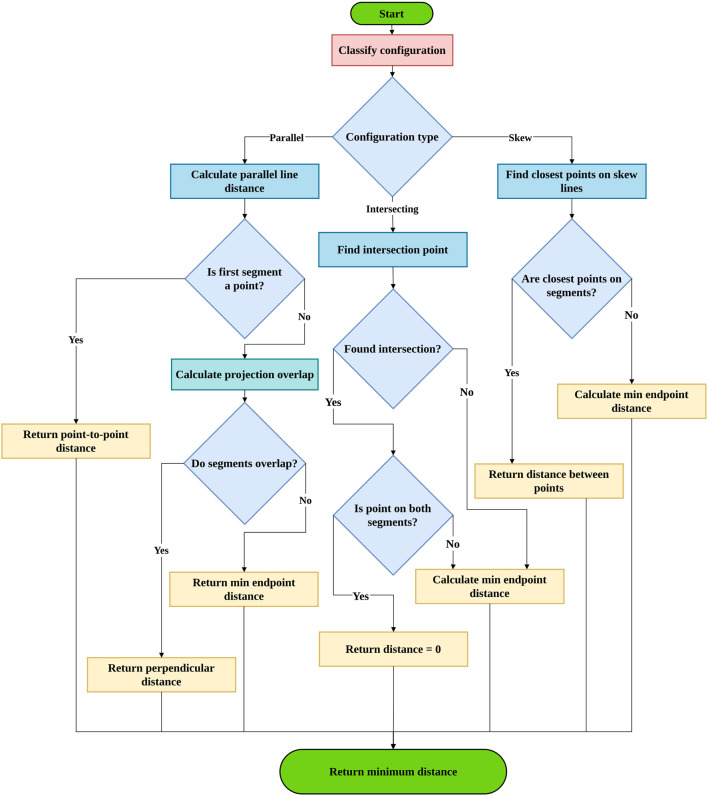
Procedure to compute minimum distance between inter-arm robot links modeled as line segments in 3-D space according to geometric configurations.

**FIGURE 4:**
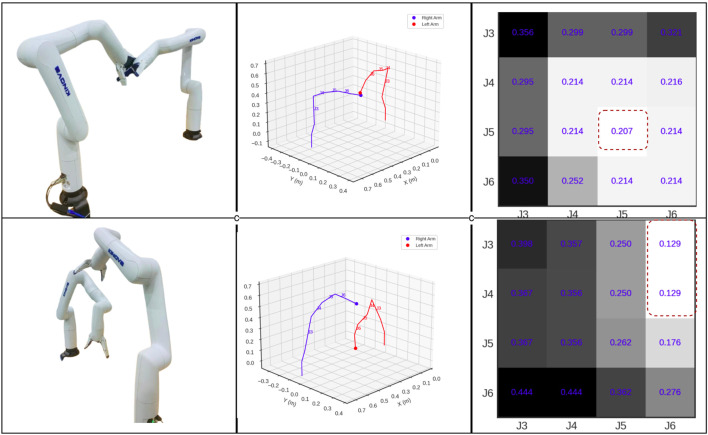
Visualization of real-time minimum distance(m) calculation for Dual-Arm Links (*block with more bright and red dotted lines indicates the minimum distance*).

The minimum distance between any pair of links in the dual-arm system is determined based on their geometric configuration, one of three cases: parallel, intersecting, or skew as in Figure 3. The method of distance calculation varies depending on the specific geometric configuration. A systematic formulation for finding the minimum distance between dual-arm links in 3-D space has been developed. Further the proposed method for calculating minimum distance is verified as shown in Figure 4. *Example Distance Calculat*ion between various cases of line segments are shown in Supplementary Material in Supplementary Figure S9.

### 3.2 Vision-based pose estimation of industrial components

Real-time asymmetric dual-arm manipulation requires rapid inference of object information within the system’s workspace. Prioritizing inference speed for effective real-time object handling, we selected the YOLOv8 OBB model for object detection, which achieved 20 frames per second on our hardware configuration while maintaining high detection accuracy. The systematic evaluation of various models used on our custom data as shown in Table 2.

**TABLE 2:** Systematic evaluation of models on our custom dataset (nut/bolt) for suitable selection of model.

S.No	Model name	Model size (MB)	Inference
1	Detectron2 Instance Segmentation	351.1	0.51
2	YOLO V8 Instance Segmentation	20.0	15
3	YOLO V11 Instance Segmentation	6.0	13
4	YOLO V8 OBB	5.9	20

#### 3.2.1 Data collection and labeling

**FIGURE 5:**
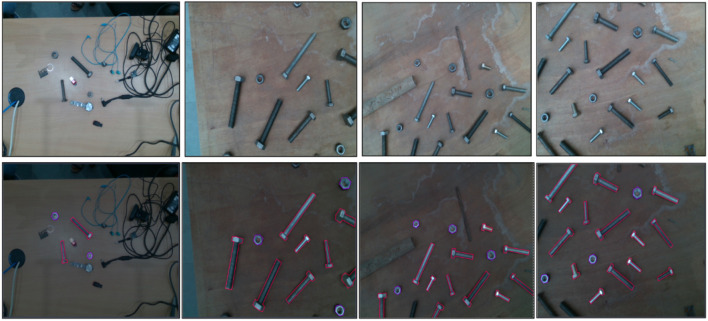
Data Collection (top row) and (b) Data Labeling of nuts (blue border) and bolts (red border) (bottom row).

Data collection and data labeling as shown in Figure 5, are crucial steps in preparing meaningful information to train deep learning models. We carefully prepared our dataset, incorporating diverse real-world conditions, including varying backgrounds (cluttered workspaces, different table surfaces), lighting, and object ages (new and old). To enhance the model’s robustness against false positives, we employed a strategic negative mining technique by adding a background/reference class. This class included objects visually similar to the target objects (bolts, nuts) and images of the empty workspace on the table. Our dataset, comprising 1,230 images, divided into training, validation, and testing sets of 1120, 80, and 30 images, respectively. Furthermore, we used data augmentation techniques of the Ultralytics training framework, increasing the dataset size tenfold. As a result, we achieved a prediction rate of 20 frames per second during model deployment, crucial for real-time applications. The system operates under controlled workspace assumptions where only target components are present, eliminating occlusion challenges typical in structured industrial sorting environments.

**FIGURE 6:**
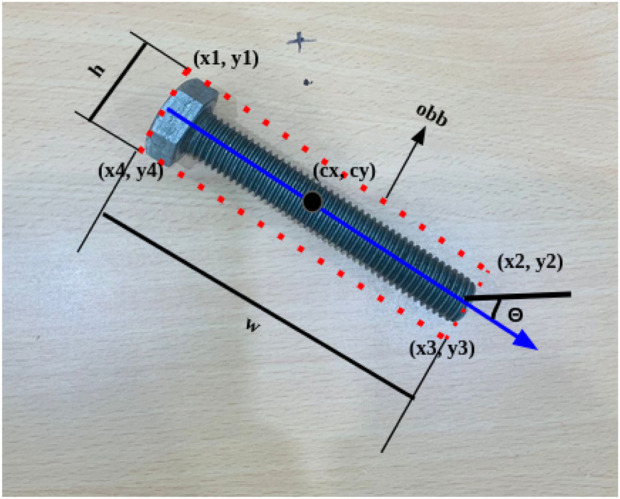
YOLOV8 Oriented bounding box predictions around the detected object.

After detecting objects (nuts and bolts) globally from an RGB-D camera, further inferred object centroids ( $c_{x}$ , $c_{y}$ ) and yaw ( θ ) along with dimensions of bounding box ( h , w ) using YOLOv8 OBB inference, as illustrated in Figure 6. Furthermore, this information is transformed into real-world coordinates from the image coordinates using projective transformations using camera intrinsic properties ( $f_{x}$ , $f_{y}$ , $C_{x}$ , $C_{y}$ ) shown below. Considering an object detected in the globally fixed camera frame, using predictions from our model is as shown in Equation 7.

$$\left[K\right]=\left[\begin{array}{ccc} f_{x} & 0 & C_{x}\\ 0 & f_{y} & C_{y}\\ 0 & 0 & 1 \end{array}\right]=\left[\begin{array}{ccc} 602.063 & 0 & 317.316\\ 0 & 602.063 & 243.314\\ 0 & 0 & 1 \end{array}\right]$$ (6)

$$F_{p}=\left[c_{x},c_{y},\theta ,h,w\right].$$ (7)

Where [K] is camera intrinsic parameter matrix of Intel Realsense2 D415, $F_{p}$ is information of features of the objects in pixels. The depth value Z for respective object’s ( $c_{x}$ , $c_{y}$ ) is obtained from the aligned depth camera. The transformation from image coordinates ( $c_{x}$ , $c_{y}$ ) to 3-D real world coordinates follows the projective transformation as shown in Equation 8.

$$Z=\frac{\text{depth}\left(c_{x},c_{y}\right)}{\gamma },\;X=\frac{c_{x}-C_{x}}{f_{x}}\cdot Z,\;Y=\frac{c_{y}-C_{y}}{f_{y}}\cdot Z.$$ (8)

γ is scaling factor, ( $C_{x}$ , $C_{y}$ ) is the principal point of the camera in pixels, $f_{x}$ , $f_{y}$ are the focal lengths in pixels, and Z is depth value in meters.

We further estimate its size using the width h of its oriented bounding box (in pixels) and angle θ , as illustrated in Equation 6 using Equation 9 and Equation 10.

$$\text{size}=\left(\frac{h}{f_{x}}\cdot Z\right)\cdot \gamma .$$ (9)

angle=θ. (10)

The final feature information of industrial components (nut/bolt) in real world coordinates are:

$$F_{mnut/bolt}=\left[X,Y,Z,\theta ,\text{size}\right]$$ (11)

### 3.3 Improved artificial potential field (iAPF) framework for dual-arm motion planning

Considering the robot position r , goal position g , other arm’s end-effector position o , and d is minimum distance between inter-arms as in Equation 5, the force fields are defined as follows:

$${\vec{f}}_{\text{att}}\left(r,g\right)=k_{a}\cdot \left(\exp\left(\left\|g-r\right\|\right)-1\right)\cdot \frac{g-r}{\left\|g-r\right\|}$$ (12)

where, $k_{a}$ is attractive force gain constant.

$${\vec{f}}_{\text{rep}}\left(r,o,d_{\min}\right)=k_{r}\cdot \frac{1}{d_{\min}^{\eta }}\cdot \frac{o-r}{\left\|o-r\right\|}$$ (13)

where $k_{r}$ is the repulsive force gain constant, η is the decay exponent, o is the obstacle position (other arm’s end-effector position), and $d_{\min}$ is the minimum distance between arm links calculated using the geometric classification method described in Section 3.1.

The home-seeking force represents a novel integration to traditional APF, providing exponential attraction towards the home position of the manipulator.

$${\vec{f}}_{\text{home}}\left(r,h\right)=k_{h}\cdot \left(\exp\left(\left\|h-r\right\|\right)-1\right)\cdot \frac{h-r}{\left\|h-r\right\|}$$ (14)

where, $k_{h}$ is the home force gain constant, h is home position defined as the predetermined starting state and safe configuration where each manipulator returns when no target objects are detected, ensuring collision-free states and optimal workspace coverage.

$${\vec{f}}_{\text{damp}}\left(v\right)=-k_{d}\cdot \vec{v}$$ (15)

where, $k_{d}$ is damping coefficient and $\vec{v}$ is current velocity.

The carefully designed force fields are crucial for achieving successful asymmetric bi-manual manipulation. The exponential goal attraction force field Equation 12, significantly enhances convergence speed and facilitates smooth transitions near the goal, essential for achieving precise pick-and-place operations. The inverse-distance repulsive force Equation 13, effectively prevents collisions between manipulators by exerting a repulsive force that scales inversely with the distance between them, ensuring robust collision avoidance while minimizing unnecessary interference. The exponential home-seeking force Equation 14, counteracts excessive arm extension, particularly in scenarios where strong repulsive forces arise due to near-goal conflicts. By encouraging the arms to return to a more neutral position, it maintains system stability and facilitates balanced convergence. Furthermore, the velocity-dependent damping force Equation 15, plays a crucial role in stabilizing the system by dissipating energy, effectively reducing the oscillatory behaviors that may arise during arm interactions, ensuring smooth and controlled trajectories.

The resultant force with damping is given by Equation 16:

$${\vec{f}}_{\text{resultant}}={\vec{f}}_{\text{att}}+{\vec{f}}_{\text{rep}}+{\vec{f}}_{\text{home}}-k_{d}\cdot \vec{v}$$ (16)

From the visual perception module described in Section 3.2, the feature information obtained in Cartesian coordinates, as defined in Equation 11, provides the position and orientation of the goal.

### 3.3.1 iAPF based linear velocities

**FIGURE 7:**
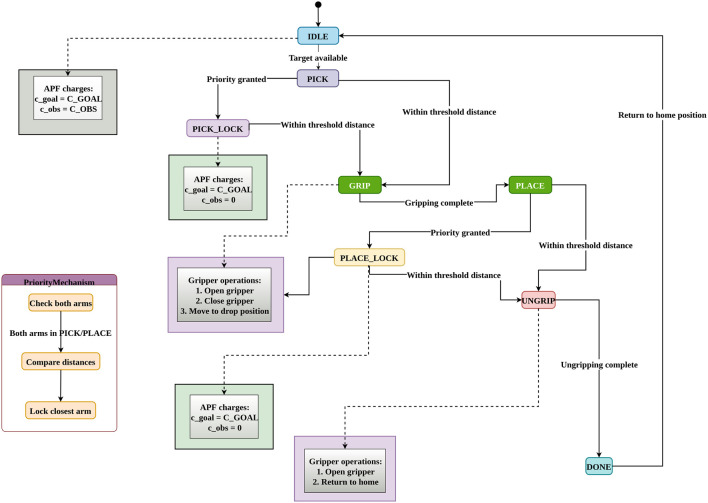
Overall states for dual-arm manipulators from picking to positioning the components.

**FIGURE 8:**
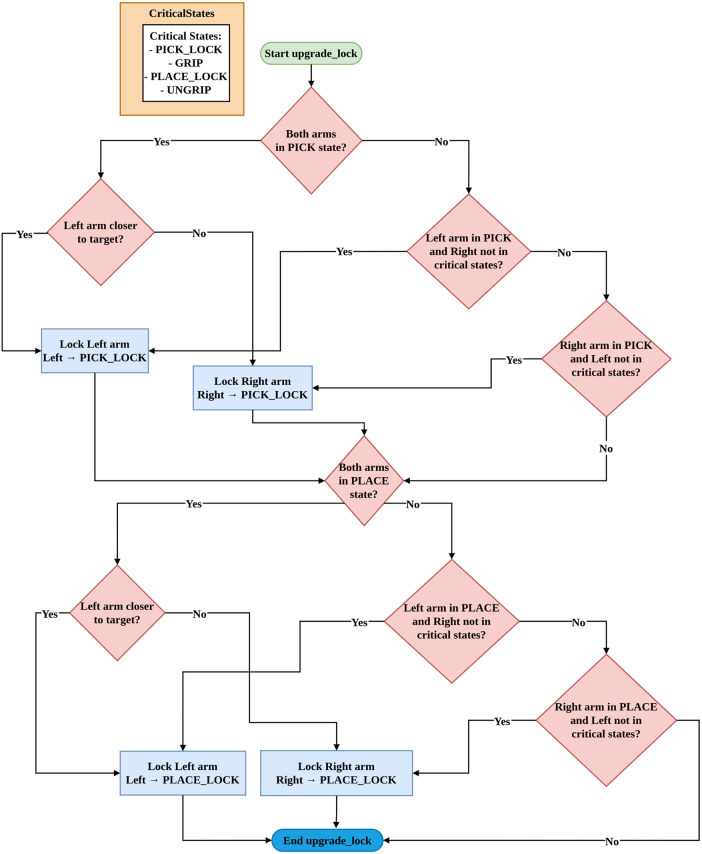
Priority mechanism and critical states for asymmetric dual-arm manipulation.

Considering the position of objects obtained from the vision module Equation 11, the generation of force fields is initiated to guide the manipulators to their respective target positions. In the absence of object detection, the target position for each manipulator defaults to its home position. The linear velocities of manipulators are determined as follows:

$${\vec{v}}_{k}=\frac{{\left({\vec{f}}_{\text{resultant}}\right)}_{k}}{s}$$ (17)

where, s is a scaling factor and k is left/right. The stability analysis for velocity based on iAPF, as defined in Equation 17, is crucial due to the non-linear nature of the potential functions. It is essential to ensure convergence to the desired position while maintaining bounded velocities. Lyapunov stability analysis shows that the superposition of attractive, repulsive, and home-seeking potentials, combined with velocity damping and state machine based priority mechanism as shown in Figures 7, 8, results in stable robot motion to the target objects.

Considering the manipulator’s end-effector position as $x\in R^{3}$ ,

$$\dot{x}=v,v=k_{v}\cdot f_{\text{resultant}}\left(x\right)$$

$$f_{\text{resultant}}\left(x\right)=-\nabla V_{\text{total}}\left(x\right)-k_{d}v.$$

The gradient $\nabla V_{\text{total}}\left(x\right)$ points the direction of steepest increase of the potential field, negative sense of this term, directs the point towards lower potential, away from obstacle and near to goal, the damping force $k_{d}v$ is subtracted from this total potential, to oppose motion proportional to velocity direction.

$$V_{\text{total}}\left(x\right)=V_{\text{att}}\left(x\right)+V_{\text{rep}}\left(x\right)+V_{\text{home}}\left(x\right),$$

where,

$$V_{\text{att}}\left(x\right)=k_{a}\left(\exp\left(\left\|x-x_{\text{goal}}\right\|\right)-1\right),$$

$$V_{\text{rep}}\left(x\right)=\frac{k_{r}}{d_{\text{obs}}^{\eta }},$$

$$V_{\text{home}}\left(x\right)=k_{h}\left(\exp\left(\left\|x-x_{\text{home}}\right\|\right)-1\right).$$

In the pre-priority state, when robots start to move towards goal, the Lyapunov function is selected as:

$$L_{1}\left(x\right)=V_{\text{total}}\left(x\right),$$

where $L_{1}\left(x\right)>0$ for all x , and $L_{1}\left(x\right)$ is continuously differentiable.

The time trajectory of $L_{1}\left(x\right)$ is given by:

$$\dot{L_{1}}\left(x\right)=\nabla V_{\text{total}}\left(x\right)\dot{x},$$

$$\dot{L_{1}}\left(x\right)=\nabla V_{\text{total}}\left(x\right)v,$$

$$\dot{L_{1}}\left(x\right)=\nabla V_{\text{total}}\left(x\right)\left[k_{v}\left({\text{f}}_{\text{att}}+{\text{f}}_{\text{rep}}+{\text{f}}_{\text{home}}-k_{d}\cdot v\right)\right].$$

At the pick-lock state ( $x=x_{\text{transition}}$ ).

Making obstacle charge $c_{\mathrm{obs}}$ and home charge $c_{\mathrm{home}}$ as zero to enable asymmetric bi-manual manipulation.

$${\text{f}}_{\text{rep}}+{\text{f}}_{\text{home}}=0.$$

Substituting these conditions, we get:

$$\dot{L_{1}}\left(x_{\text{transition}}\right)=\nabla V_{\text{att}}\left(x\right)\left[k_{v}\left({\text{f}}_{\text{att}}-k_{d}\cdot v\right)\right].$$

The time trajectory at $x_{\text{transition}}$ indicates potential for further movement.

$$\dot{L_{1}}\left(x_{\text{transition}}\right)=\nabla V_{\text{att}}\left(x\right)\left[k_{v}\left(-\nabla V_{\text{att}}\left(x\right)-2k_{d}\cdot v\right)\right].$$

Simplifying, we have:

$$\dot{L_{1}}\left(x_{\text{transition}}\right)=-k_{v}\|\nabla V_{\text{att}}\left(x\right){\|}^{2}-2k_{v}k_{d}\nabla V_{\text{att}}\left(x\right)\cdot v.$$

For $k_{d}>0$ and $k_{v}>0$ , the following holds:

$$\dot{L_{1}}\left(x_{\text{transition}}\right)<0\;\text{for all }x\neq x_{\text{goal}},$$

$$\dot{L_{1}}\left(x_{\text{transition}}\right)=0\;\text{for }x=x_{\text{goal}}.$$

The stability analysis demonstrates, dual-arm iAPF control achieves coordinated manipulation through two key phases. Initially, both manipulators move under complete force set (attractive, repulsive, home-seeking) with Lyapunov function $L_{1}\left(x\right)$ ensuring stable concurrent motion. When priority is assigned based on goal distance, the system transitions to a reduced force set (attractive and damping only) for the prioritized arm, with $L_{1}\left(x\right)$ guaranteeing asymptotic stability to the goal position. The non-zero $\dot{L_{1}}\left(x\right)$ at transition points enables sequential task completion while maintaining collision avoidance, validating the proposed approach’s theoretical stability and practical effectiveness for industrial manipulation tasks.

### 3.3.2 PD controller based angular velocities

Considering the orientation of objects obtained from the vision module, the current end-effector orientation for each manipulator is obtained through real-time forward kinematics of the dual-arm system, providing the basis for orientation control.

The current orientation of the end-effector is given by:

$$\theta_{c}=\left[\theta_{cx},\theta_{cy},\theta_{cz}\right]\in SO\left(3\right)$$

The desired orientation, obtained from visual perception given by:

$$\theta_{d}=\left[\theta_{dx},\theta_{dy},\theta_{dz}\right]\in SO\left(3\right)$$

The corresponding rotation matrix is represented as:

$$R\left(\theta \right)=R_{z}\left(\theta_{z}\right)R_{y}\left(\theta_{y}\right)R_{x}\left(\theta_{x}\right)$$

The error rotation matrix is given by:

$$R_{e}=R_{d}R_{c}^{-1}\in SO\left(3\right)$$

The error rotation matrix $R_{e}$ can be expressed in the axis-angle representation, parameterized by angle θ and axis $k=\left[k_{x},k_{y},k_{z}\right]$ .

The PD control law for orientation control of manipulator given by:

$$\omega_{k}=K_{p}\cdot \theta \cdot k+K_{d}\cdot \frac{d}{dt}\left(\theta \cdot k\right)$$ (18)

Where $K_{p}\;\in R^{3\times 3}$ is the proportional gain matrix, $K_{d}\;\in R^{3\times 3}$ is the derivative gain matrix, $\omega \in R^{3}$ is angular velocity vector and θ∈R is the rotation angle error derived from the error rotation matrix $R_{e}$ .

## 4 Experimental setup

**FIGURE 9:**
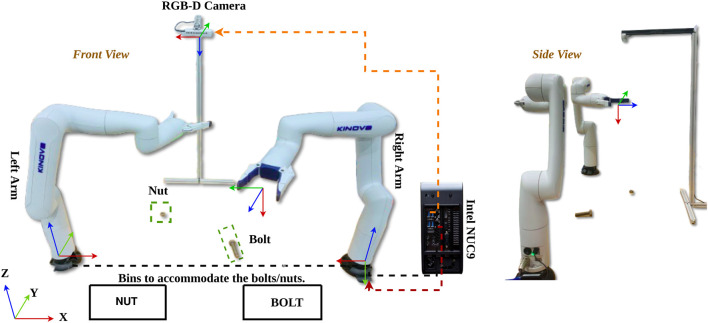
Experiment setup to demonstrate the iAPF based motion planning for dul-arm asymmetric manipulations.

The experimental setup for demonstrating asymmetric dual-arm manipulation is established with reference frames as shown in Figure 9. The system comprises two 6-DOF Kinova Gen3 Lite arms mounted on a fixed table, with an overhead Realsense2 D415 camera providing a global view of the workspace. Processing is handled by an Intel Nuc9I7Qnx with 32 GB RAM, which connects to the manipulators via TCP/IP sockets using a specific communication protocol, while the camera interfaces through USB. The Kinova-Kortex Python API is used for manipulator control, and the ROS-Noetic framework facilitates publishing and subscribing to the vision module’s inference data.

Individual forward kinematics, as given by Equation 4, provide the position and orientation of each manipulator’s end-effector with respect to its respective base frame. Furthermore, $T_{c}^{r}$ represents the transformation of the camera in the right arm’s base frame, while $T_{r}^{l}$ denotes the transformation of the right arm in the left arm’s base frame.

$$T_{c}^{r}=\left[\begin{array}{cccc} 1 & 0 & 0 & 0.251\\ 0 & -1 & 0 & -0.211\\ 0 & 0 & -1 & 0.75\\ 0 & 0 & 0 & 1.0 \end{array}\right]T_{r}^{l}=\left[\begin{array}{cccc} -1 & 0 & 0 & L\\ 0 & -1 & 0 & 0.0\\ 0 & 0 & 1 & 0.0\\ 0 & 0 & 0 & 1.0 \end{array}\right]$$

The further pose from Equation 11, is transformed to the left arm’s base as Equation 19:

$$p_{i}^{l}=T_{r}^{l}T_{c}^{r}p_{i}^{c}$$ (19)

where $p_{i}^{c}$ is given by:

$$p_{i}^{c}={\left[\begin{array}{cccc} X_{i}^{c} & Y_{i}^{c} & Z_{i}^{c} & 1.0 \end{array}\right]}^{T}$$

The rotation angle $\theta_{i}$ is computed as:

$$\theta_{i}=\theta_{c_{i}}\cdot \frac{180}{\pi }$$

The gripper angle $\theta_{{\text{gripper}}_{k}}$ for the arm is given by Equation 20:

$$\theta_{{\text{gripper}}_{k}}=270-\theta_{i}$$ (20)

This ensures proper alignment between the gripper and nut/bolt for grasping.

Both linear velocities (from iAPF) and angular velocities (from PD control) for both arms are initially calculated in left base frame using Equation 17 and Equation 18. Subsequently, the right arm velocities (both linear and angular) are transformed from left base frame to right base frame using $T_{l}^{r}$ rotation matrix, while left arm velocities remain in left base frame as shown in Equation 21.

$$v_{r}=R_{l}^{r}\cdot {\left(v_{r}\right)}_{l};\;\omega_{r}=R_{l}^{r}\cdot {\left(\omega_{r}\right)}_{l};\;v_{l}=v_{l};\;\omega_{l}=\omega_{l}.$$ (21)

Where i represents the nut/bolt, k denotes the right/left arm, $R_{l}^{r}$ is rotation matrix of $T_{l}^{r}$ , ${\left(v_{r}\right)}_{l},{\left(\omega_{r}\right)}_{l}$ are linear and angular velocities of right arm in left base and $v_{r},\omega_{r}$ are transformed velocities in right base frame.

## 5 Results and discussion

To validate the proposed system for asymmetric dual-arm manipulation framework for sorting industrial components, created challenging scenarios by placing nuts and bolts in close proximity, deliberately inducing goal conflicts between the manipulators. This setup rigorously tests the framework’s collision avoidance capabilities, priority-based state transitions, and the effectiveness of exponential attractive home-seeking forces in maintaining stable coordination during sorting tasks.

The performance metrics for the bolt and nut detection classes demonstrate superior discrimination capabilities compared to the reference class. Bolts achieve 0.99 detection accuracy and nuts achieve 0.97 detection accuracy, with minimal inter-class confusion of 0.02. The F1 score reaches 0.89 at a confidence threshold of 0.120, while precision achieves 1.00 at 0.808. Recall maintains 1.00 at low confidence thresholds, indicating robust detection even under varying conditions. These metrics validate the model’s effectiveness in discriminating between nuts and bolts while successfully handling the reference class for false positive reduction. The final deployment results are as shown in Deep Learning Model Metrics and Real-Time Vision Update Experiment Figures are shown in Supplementary Figures S1-S8.

**FIGURE 10:**
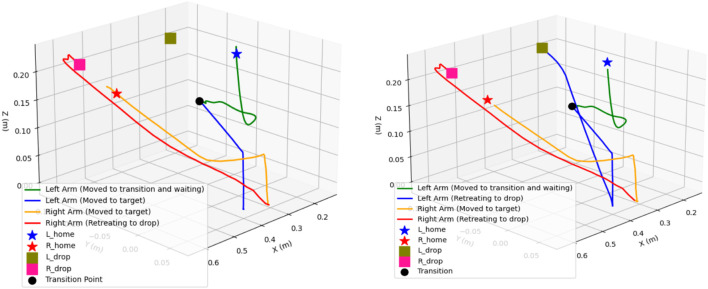
Dual-arm end effectors’ trajectories with exponential attraction, inverse distance and exponential home-seeking attraction force fields. [Both arms initiate movement from their home positions towards their respective target locations. Due to distance priority, the right arm reaches its target first, picks up the object, and then retreats to its designated drop point. While the right arm is performing these actions, the left arm remains at the transition point. Once the right arm has move away by picking the object, the left arm begins its movement towards its target. The second plot illustrates the complete trajectories of both arms].

The proposed iAPF, incorporating three distinct force fields, effectively addressed near-goal conflict scenarios, as demonstrated in Figure 10. The implementation of three force fields: exponential goal attraction, inverse-distance other-arm repulsion, and home exponential attraction, demonstrates superior control over dual-arm trajectories, effectively mitigating inter-arm collision. The transition point shows deliberate stop and stable movement of the arms during collision possible scenarios and maintains better spatial separation throughout their movements. The exponential home attraction force acts as a regulator, preventing excessive arm extension in scenarios where near-goal conflicts result in strong repulsive forces. The establishment of a three-way force equilibrium results in more predictable and controlled motion paths. The home force establishes natural boundaries for arm movements, maintaining optimal manipulator poses and preventing over-extension. Particularly, how the trajectories exhibit smoother curves with minimal oscillations, as the arms move toward goals, the exponential home attraction scales with distance, providing graduated control that keeps configurations within safe operating ranges. This makes the system more robust against kinematic singularities while ensuring efficient task completion.

**FIGURE 11:**
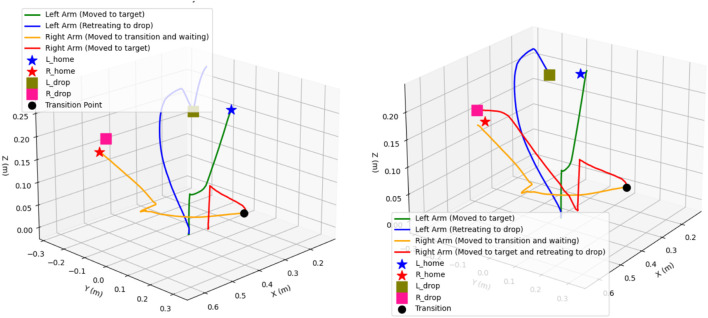
Dual-arm end effectors’ trajectories with exponential attraction, inverse distance repulsion without exponential home-seeking attraction force fields. [Both arms initiate movement from their home positions towards their respective target locations. Due to distance priority, the left arm reaches its target first, picks up the object, and then retreats to its designated drop point. While right arm remains at the transition point. Once the left arm has move away by picking the object, the right arm begins its movement towards it target. The second plot illustrates the complete trajectories of both arms].

In traditional attractive-repulsive APF, as shown in Figure 11 without home attraction, the system exhibits inherent instabilities near goal regions. While the arms successfully navigate to their target locations, the trajectories demonstrate less controlled movements with larger sweeping motions and more aggressive approaches. The fundamental issue lies in the force imbalance - as both arms approach their respective goals simultaneously, the attractive forces dominate while the inter-arm distance decreases, causing a sudden spike in repulsive forces. This force antagonism leads to oscillatory behaviors and potential over-extension of the arms. Without a stabilizing home force, the arms can reach configurations near kinematic singularities with no natural mechanism to recover optimal poses. The problem is particularly pronounced when both arms operate in close proximity, where the rapid transition between attraction-dominated and repulsion-dominated states can lead to unstable motion patterns.

**FIGURE 12:**
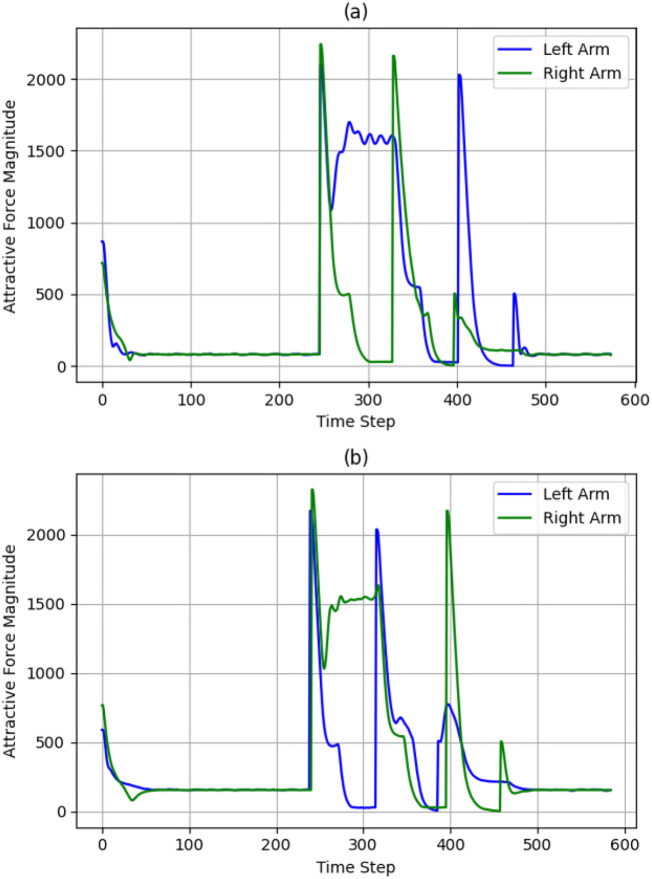
Convergence of attractive forces for both cases **(a)** with exponential home **(b)** without exponential home.

**FIGURE 13:**
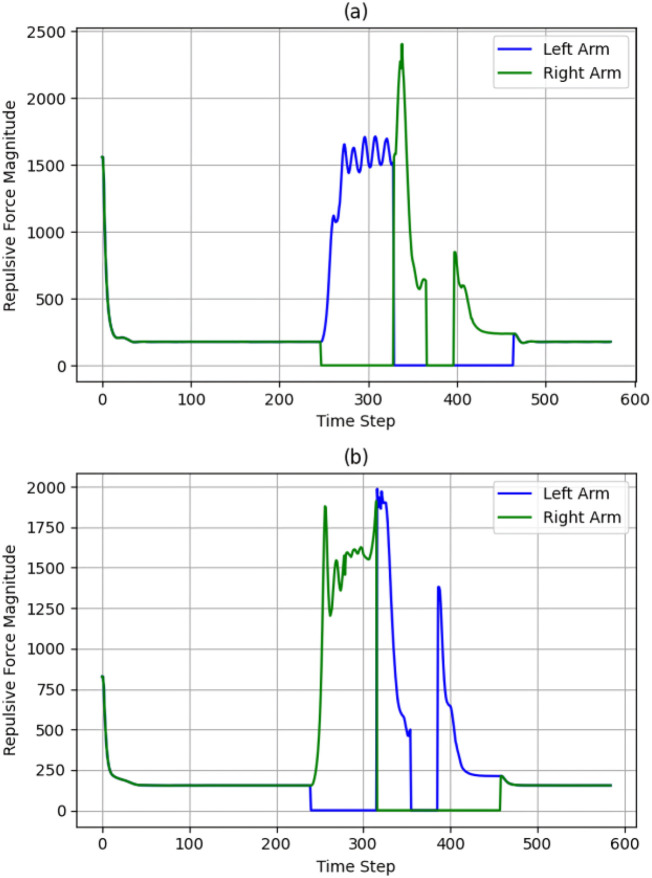
Convergence of Repulsive forces for both cases Forces **(a)** with exponential home **(b)** without exponential home.

Observing Figures 12a, 13a, 10, represents attractive and repulsive force trends for our iAPF which represents three-way force equilibrium: Both arms exhibit controlled convergence to home positions with initial attractive forces of ≈ 900 (left) and ≈ 700 (right) stabilizing to ≈ 50 baseline, demonstrating effective home-seeking behavior before vision activation. Upon target detection at t = 248, right arm’s ≈ 2400, left arm’s ≈ 2100 attractive and ≈ 1500 oscillating repulsive forces until t = 330 show initial target approach with active collision avoidance. Right arm then dominates with ≈ 2300 attractive peak at t = 350 while left arm maintains oscillating pattern around ≈ 1600 before dropping to ≈ 0 at t = 388, indicating priority-based sequential execution. The left arm’s subsequent ≈ 2000 spike at t = 420 and final ≈ 500 adjustment before ≈ 50 convergence, combined with right arm’s ≈ 2400-to-zero repulsive transition and ≈ 700 adjustment spike, demonstrate the three-way force equilibrium effectively managing collision avoidance, target acquisition, and stability maintenance. These coordinated force patterns enable safe dual-arm manipulation through balanced home-seeking, exponential attraction, and inverse-square repulsion, validated by smooth transitions, predictable force scaling, and clear trajectory-force correlations.

Observing Figures 12b, 13b, 11, analyzing the second row sub-plots represents attractive and repulsive force trends for traditional attractive-repulsive APF which represents two-way force equilibrium: Both arms show initial convergence to home position with ≈ 550 (left) and ≈ 700 (right) attractive force stabilizing to ≈ 200 until around t = 248, showing weaker position holding without home-seeking force. After target detection from vision, simultaneous high-magnitude attractive forces emerge ( ≈ 2300 right, ≈ 2100 left) with ≈ 1900 repulsive force oscillations, indicating uncontrolled collision avoidance. Both arms exhibit competing behavior during t = 300–350, with right arm’s ≈ 1600 attractive spike concurrent with left arm’s ≈ 2000 peak, leading to unstable spatial competition. The force equilibrium deteriorates with sharp ≈ 2000 repulsive spikes and minimal damping, causing wide trajectory deviations visible in the path curves. Final phase shows multiple high-magnitude force oscillations before eventual convergence to 200 baseline, demonstrating poor stability without home-seeking influence. The trajectory plot validates these issues through excessive path curvature, wider sweeping motions during collision avoidance, and less direct approaches to targets, confirming the necessity of home-seeking force for stable dual-arm coordination.

**FIGURE 14:**
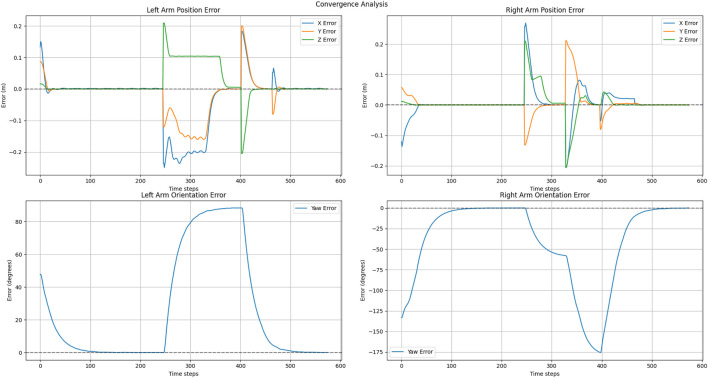
Position and orientation convergence in exponential goal attraction, exponential home attraction and inverse distance repulsion.

**FIGURE 15:**
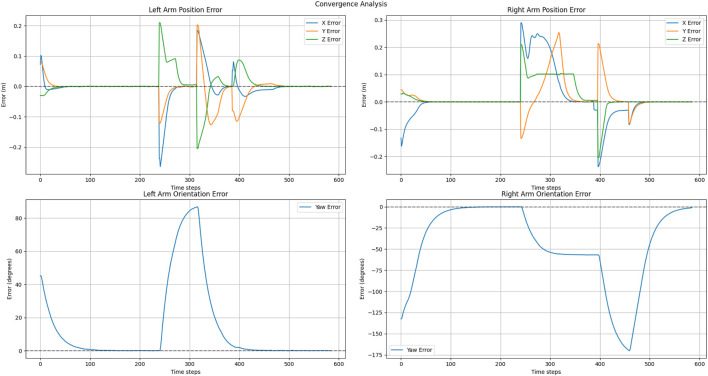
Position and orientation convergence in exponential goal attraction, without exponential home attraction and inverse distance repulsion.

**FIGURE 16:**
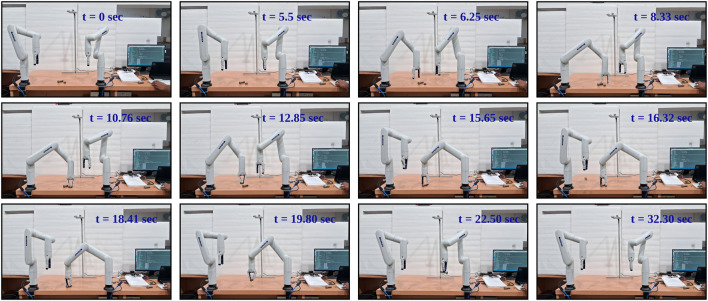
Sequential Illustration of Asymmetric Dual-Arm Manipulation at Near Goal Conflict situation with Clear Spatial Separation between Arms [at t = 6.25 left arm gets priority due to the proximity, t = 8.33 left arm picking the object while right arm waiting at safe transition point, t = 15.66 right arm picks the object and t = 16.32 left arm drops the object and right arm at t = 22.50.] - With Three-way Force Equilibrium.

**FIGURE 17:**
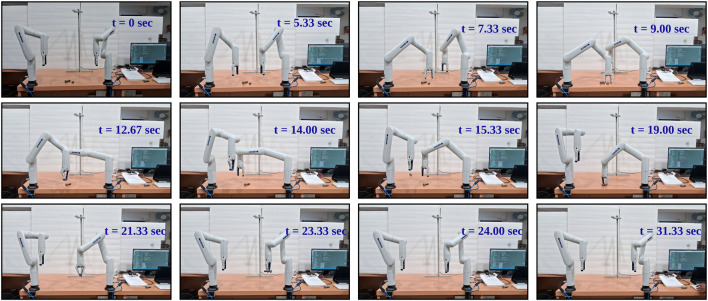
Sequential Illustration of Asymmetric Dual-Arm Manipulation at Near Goal Conflict situation with Close Proximity Movement of Dual-Arm [at t = 7.33 left arm gets priority due proximity with goal, while right arm move far away from left arm due to spike in repulsion, t = 14 arms are near collision situation.] - Without Three-Way Force Equilibrium.

The convergence behaviors depicted in Figures 14, 15, illustrate the interplay of two parallel control systems. Position trajectories are governed by the APF framework, while orientation trajectories are controlled by a PD controller. Figure 14 demonstrates smooth convergence, attributed to the inclusion of the three-way force equilibrium that effectively mitigates oscillations and prevents excessive arm extension. Notably, orientation convergence is independently controlled and effectively synchronized with position convergence. But in Figure 15 shows that without home attraction, position trajectories oscillate due to force antagonism between pure attraction-repulsion, while orientation still achieves convergence with PD control but experiences coupling effects from less stable positional behavior. This separation of position and orientation control allows for independent tuning of linear and angular responses while maintaining overall system stability. The sequential illustration of experiments for explaining two cases are shown in Figures 16, 17.

## 6 Conclusion

In this paper, we presented a comprehensive framework for dual-arm asymmetric manipulation with inter-arm collision avoidance for handling industrial components. Our approach introduces a computationally efficient collision detection method that represents manipulator links as line segments in 3-D space, enabling real-time distance monitoring through analytical solutions for parallel, intersecting, and skew configurations. The integration of YOLOv8 OBB-based object detection achieves robust real-time perception at 20 frames per second with oriented bounding boxes, demonstrating high accuracy of 0.99 and 0.97 for bolt and nut detection respectively along with components size estimation. The improved Artificial Potential Field (iAPF) framework implements a novel three-way force equilibrium through exponential attractive, inverse-square repulsive, and exponential home-seeking forces, significantly enhancing trajectory stability and reducing oscillations compared to traditional APF approaches. Our hybrid twist control scheme combines iAPF-generated linear velocities with PD-controlled angular velocities, with proven stability through Lyapunov analysis and enabling precise asymmetric manipulation through a priority-based state machine. Experimental validation demonstrates the framework’s effectiveness in handling challenging scenarios, including close-proximity object sorting and goal conflicts between manipulators, while maintaining safe separation distances. This integrated approach provides a promising foundation for deploying collaborative dual-arm systems in industrial settings where reliable, efficient, and safe manipulation of industrial components is essential. The proposed framework assumes a controlled workspace free from external dynamic obstacles, focusing primarily on inter-arm collision avoidance. The vision system is currently limited to pre-trained component classes (nuts/bolts), though it remains adaptable through retraining for new object types.

Future work will extend the present framework to handle external dynamic environments through: i) real-time external obstacle detection using RGB-D depth sensing to detect unknown objects in the workspace, ii) adaptive repulsive force field generation that incorporates external obstacles into the existing iAPF framework. This progression will enable deployment in unstructured manufacturing environments while maintaining the proven stability and collision avoidance capabilities demonstrated in this work. Potential applications include automated assembly of industrial components, transitioning from absolute motion to relative motion control during assembly phase, with integrated size matching and precise alignment capabilities.

## Data Availability

The datasets and codes generated/analyzed for this study can be found in the Dual-Arm-Manipulation, https://github.com/suryarobotcontrol/Dual-Arm-Manipulation.git.
