# Supplementary material for: iAPF: an improved artificial potential field framework for asymmetric dual-arm manipulation with real-time inter-arm collision avoidance
Source: Front Robot AI. 2025 Oct 28;12:1604506. doi: 10.3389/frobt.2025.1604506 (PMC12602476; doi:10.3389/frobt.2025.1604506)
Supplement: Supplementary file 1 [file Supplementaryfile1.pdf]

Supplementary Material

1 SUPPLEMENTARY DATA

2 SUPPLEMENTARY TABLES AND FIGURES

2.1 Figures

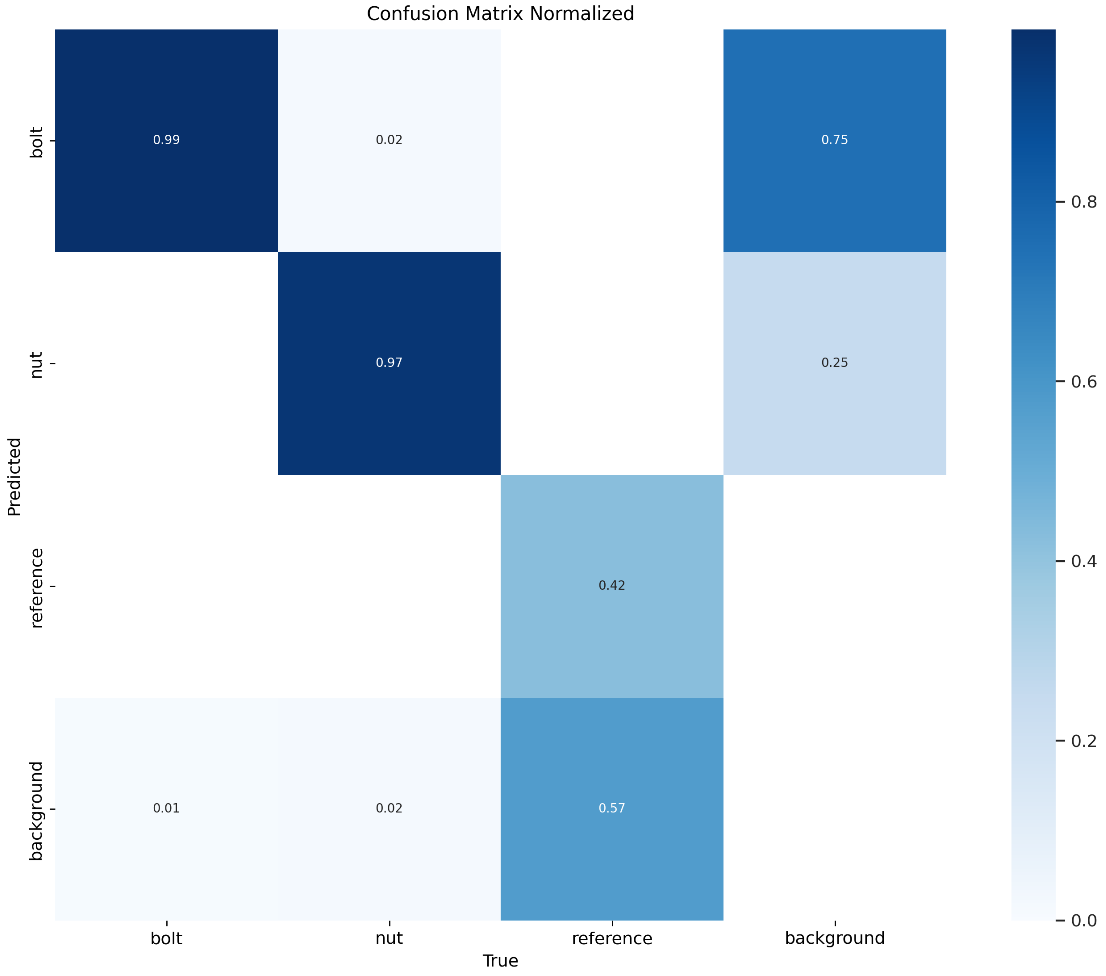

Figure S1. Bolts: 0.99 detection accuracy, Nuts: 0.97 detection accuracy, and Inter-class confusion of only 0.02, indicating strong discrimination

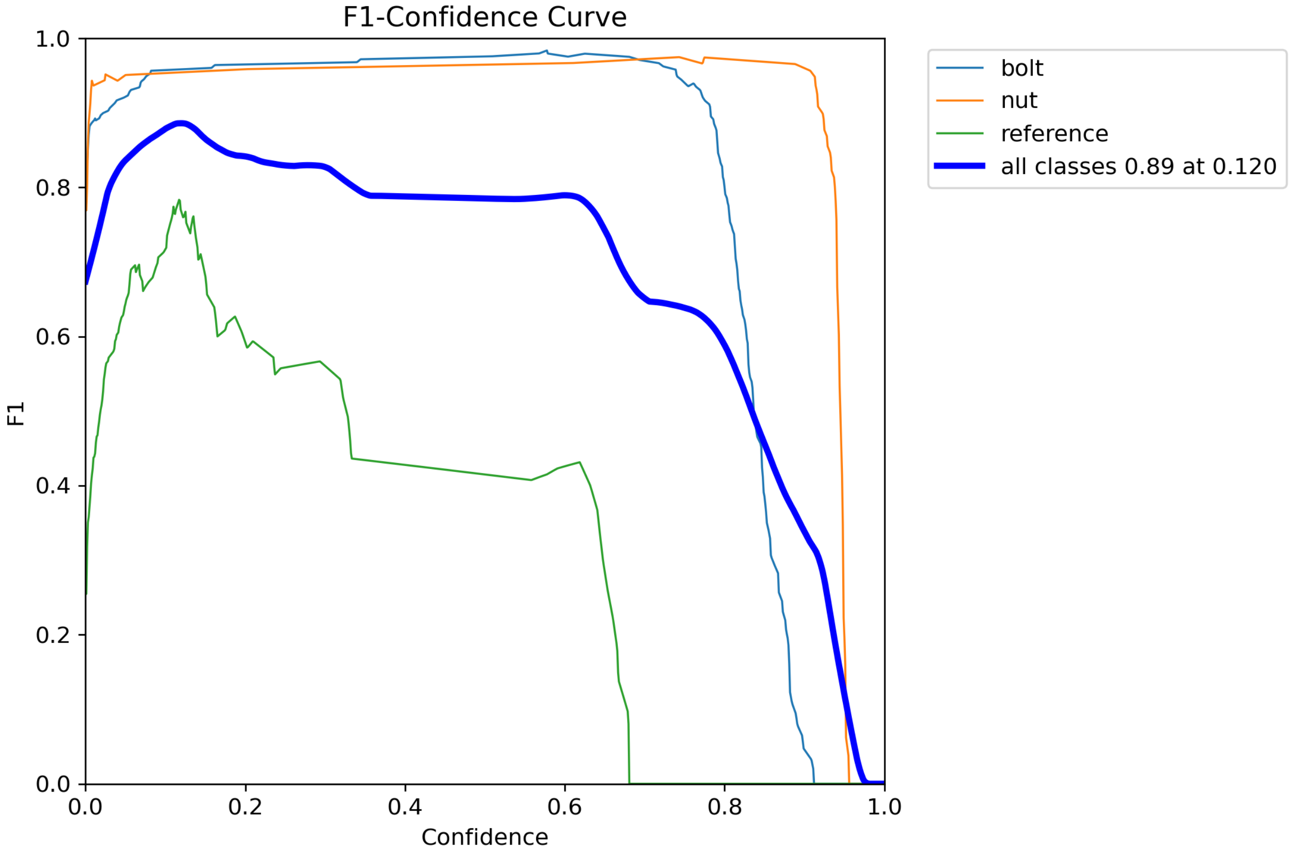

Figure S2. F1 score reaches 0.89 at confidence threshold 0.120

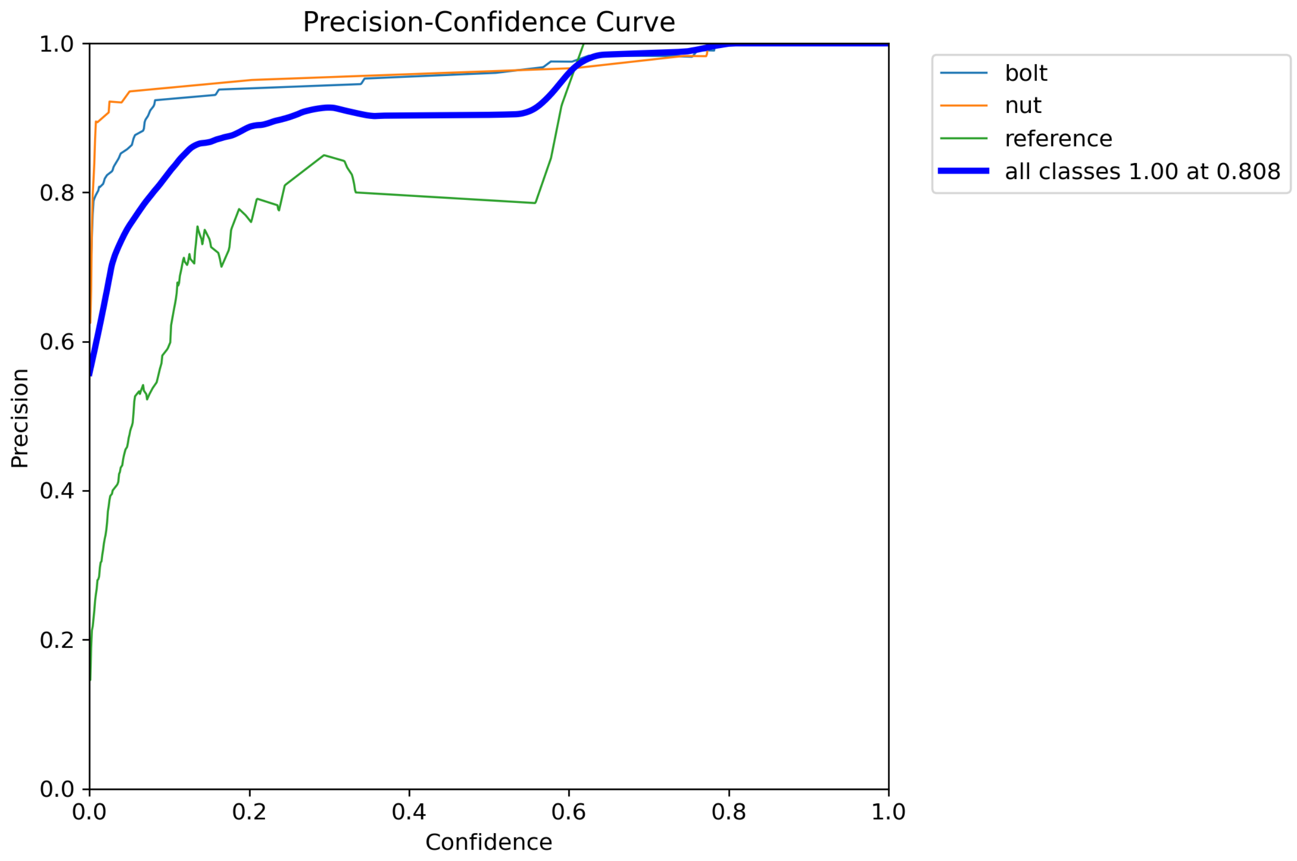

Figure S3. Precision achieves 1.00 at 0.808

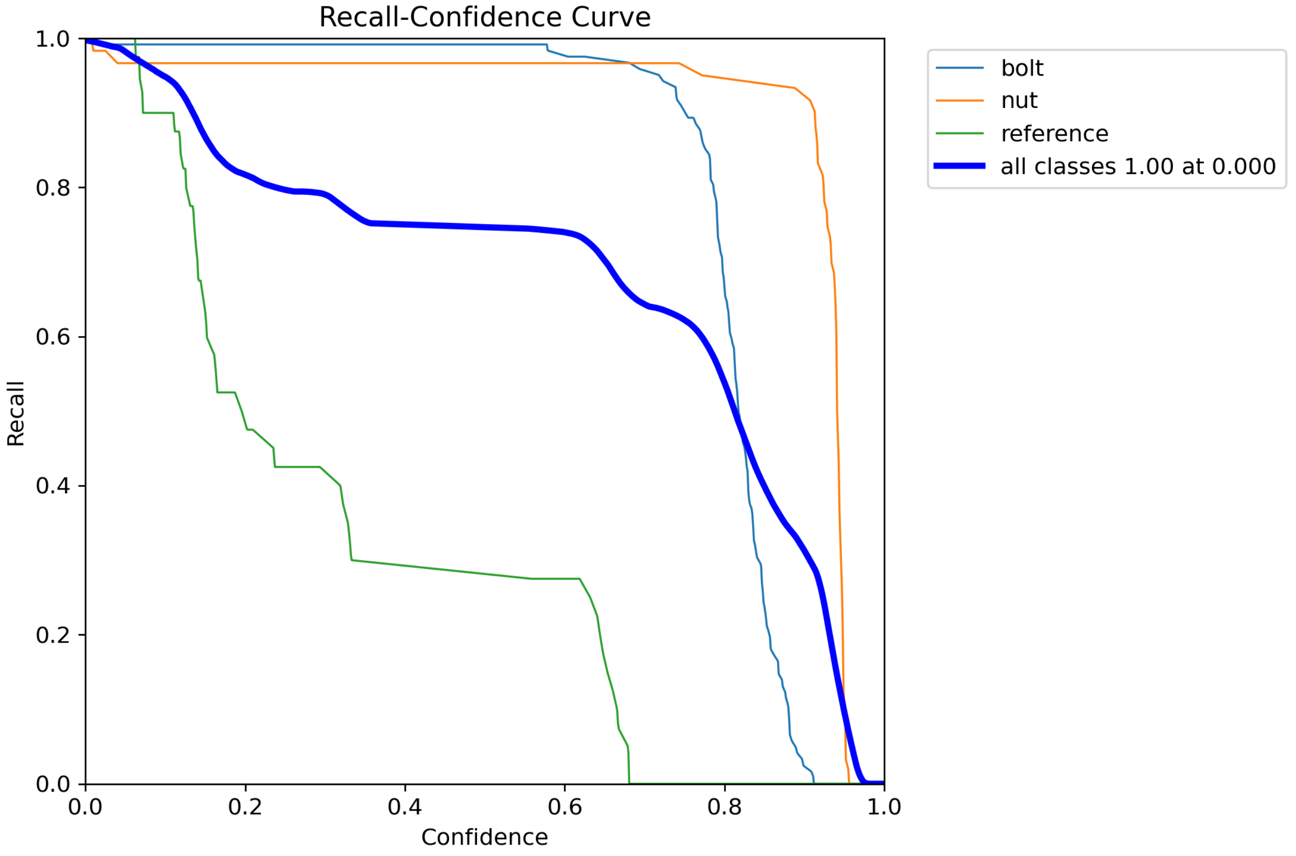

Figure S4. Recall maintains 1.00 at low confidence thresholds.

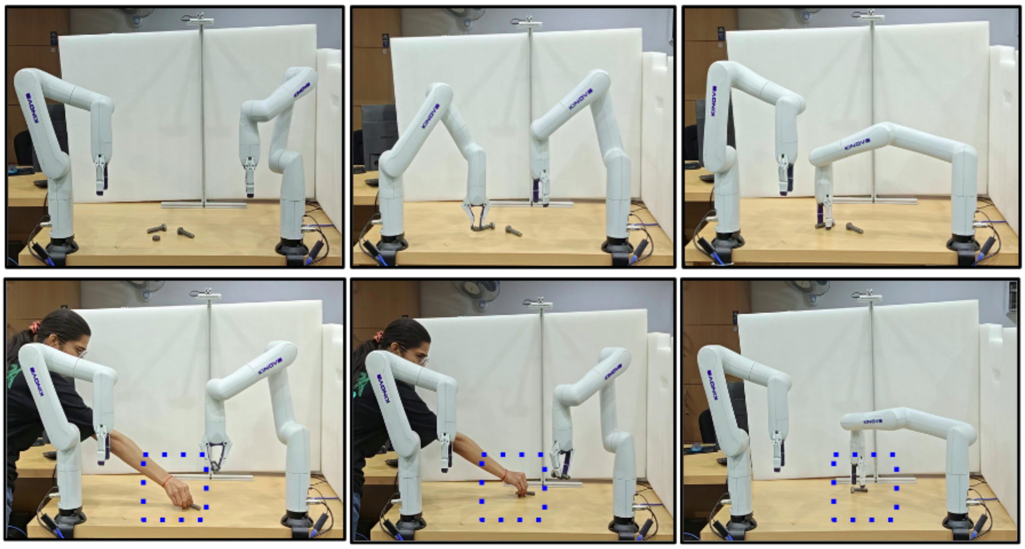

Figure S5. Real-Time Vision Update for Dual-Arm Manipulation Experiment [Upon changing the position of the object, updated pose the object in communicated to the corresponding arm in real-time]

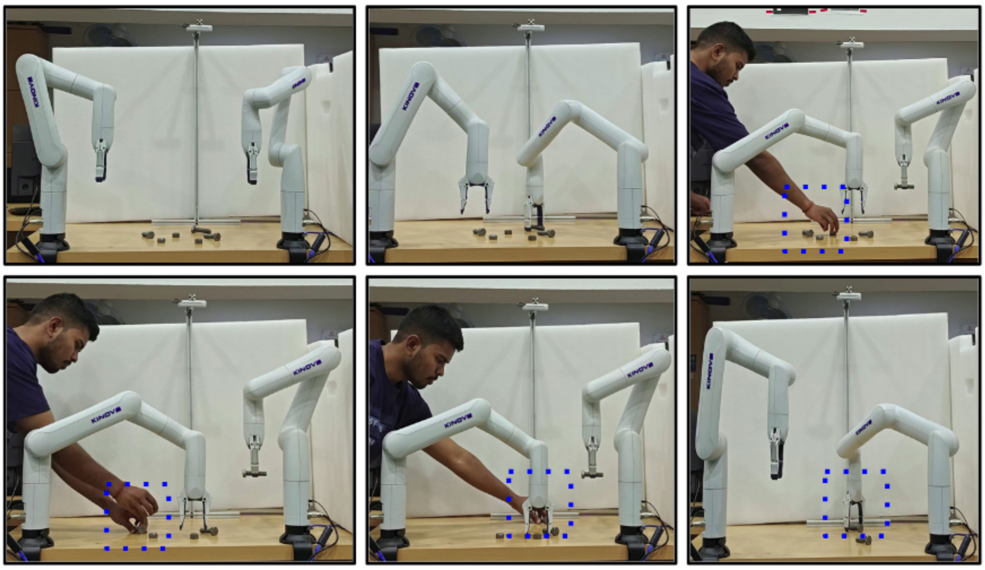

Figure S6. Real-Time Vision Update for Dual-Arm Manipulation Experiment [Upon changing the position of the object, updated pose the object in communicated to the corresponding arm in real-time].

Figure S7. Real-Time Vision Update. **(S5)** Experiment 1 **(S6)** Experiment 2.

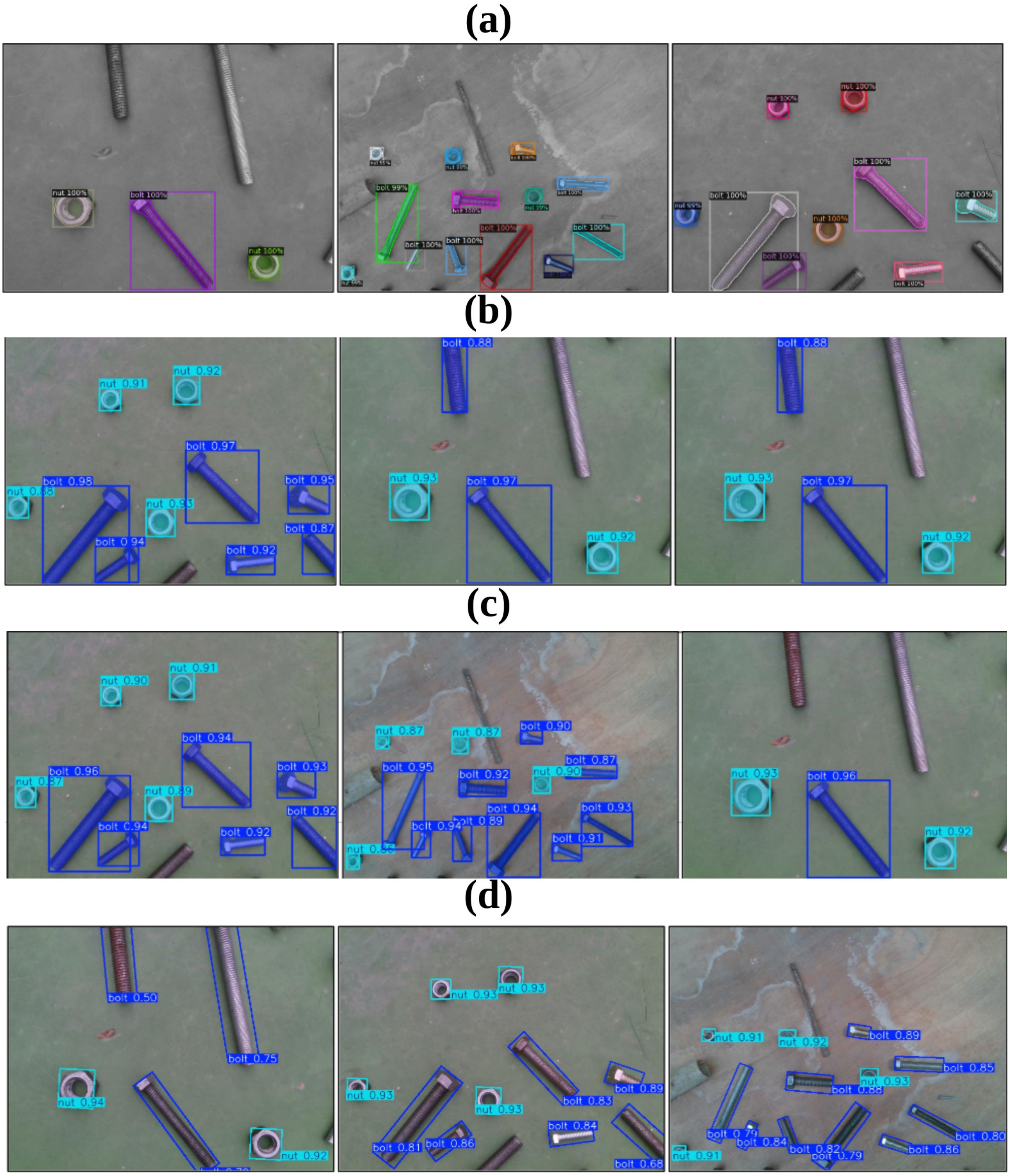

Figure S8. Predictions from (a) Detectron2 (b) YOLOV8 (c) YOLOV11 models trained on our custom-daset of nuts and bolts segmentation and bounding box (d) YOLOV8 obb.

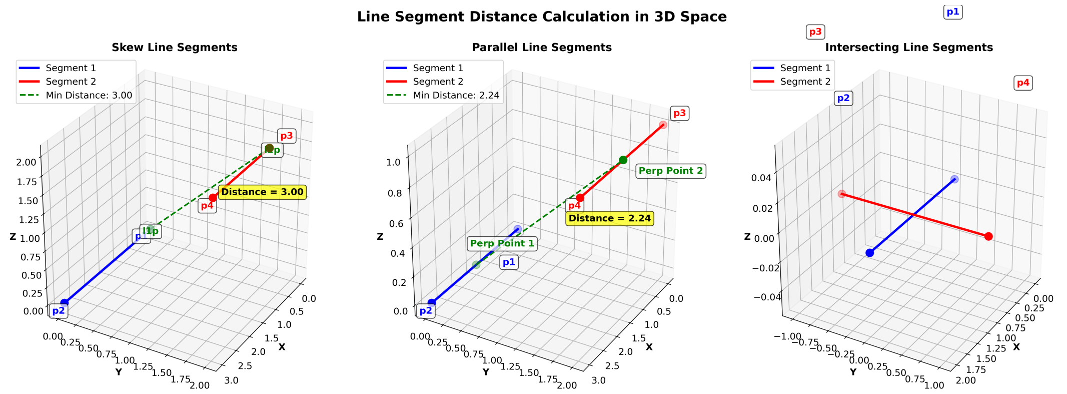

Figure S9. Minimum distance between different configuration line segments with([p1][p2][p3][p4]) (a) Skew line segments ([0, 0, 0][3, 0, 0][1, 2, 2][3, 2, 2]) (b) Parallel line segments ([0, 0, 0][3, 0, 0][0, 2, 1][3, 2, 1]) (c) Intersecting line segments ([0, 0, 0][2, 0, 0][1, -1, 0][1, 1, 0])
